# How safe from *Listeria monocytogenes* are frozen ready-to-eat fish products, either cooked or smoked, when stored under refrigeration after purchasing?

**DOI:** 10.1007/s42770-026-02103-7

**Published:** 2026-10-05

**Authors:** Mayara S. Tavares, Patrícia G. Paulino, Daniele S. Juliano, Roberto Laureano-Melo, Huarrisson A. Santos, Rosa H. Luchese

**Affiliations:** 1 Food Microbiology Laboratory, Department of Food Technology, Federal Rural University of Rio de Janeiro, 23.897-970 Seropédica, Rio de Janeiro, Brazil; 2 Poultry Health Laboratory, Department of Epidemiology and Public Health, Federal Rural University of Rio de Janeiro, 23.897-970 Seropédica, Rio de Janeiro, Brazil; 3 Department of Physiological Sciences, Institute of Biological and Health Sciences Federal Rural University of Rio de Janeiro, Seropédica, Rio de Janeiro, 23897000 Brazil

**Keywords:** Kani-kama, Smoked salmon, Background microbiota, Lactic acid bacteria, *Listeria* sp

## Abstract

This research aimed to access the incidence of *Listeria* sp. and *L. monocytogenes* in frozen ready-to-eat (RTE) fish products immediately after purchasing, and after refrigerated storage. Out of 72 kani-kama samples, 17 (23,6%) and 16 out of 48 samples (33,3%) of smoked salmon samples, tested positive for *Listeria* sp. by the traditional culture method, whereas 45,8% and 56.2% were positive by PCR, for kani-kama and smoked salmon samples, respectively. Applying qPCR, 21 samples of kani-kama out of 72 (29.2%) and 23 out of 48 samples of smoked and sliced salmon (47.9%) were positive for *L. monocytogenes*. Quantification was performed by culture to account only for viable cells, and confirmation was carried out via PCR and qPCR for *Listeria* sp. and *L. monocytogenes*, respectively. The average counts of *L. monocytogenes* did not exceed 2 Log CFU/g, both in kani-kama or smoked salmon. However, 15.3% of individual kani-kama samples and 14,6% of smoked salmon samples showed counts of up to 3.2 Log CFU/g, some even immediately after purchasing. Higher number of positive samples was observed during refrigerated storage compared to the number of samples analysed immediately after purchasing, indicating that refrigeration for a long time can pose an additional health threat. Unlike kani-kama, which is a heat-processed product, a higher background microbiota count was detected in smoked salmon. However, this background microbiota apparently did not influence the growth of *Listeria* spp.

**Supplementary Information:**

The online version contains supplementary material available at https://doi.org/10.1007/s42770-026-02103-7.

## Introduction

There is a growing demand for refrigerated fresh ready-to-eat (RTE) foods and microbiological criteria for both national and international trade are becoming more stringent [1, 2]. The RTE foods that are expected to have near neutral or moderately low pH and relatively high-water activity (a_w_) values are considered able to support the growth of *L. monocytogenes*, an important psychrotrophic foodborn pathogen. The RTE surimi kani-kama and smoked salmon fall within these characteristics and therefore can support the growth of *L. monocytogenes*. According to the zoonoses monitoring activities carried out in 2021 by the European Food Safety Authority and the European Centre for Disease Prevention and Control, listeriosis is the fourth most reported zoonosis and together with West Nile virus infections were the most severe zoonotic diseases, with highest fatality rates. The highest recorded occurrence of *L. monocytogenes* was in meat RTE followed by fish RTE [3].

A tolerance limit of 100 CFU/g was established by Brazilian Normative Instruction nº 161 [4] for RTE foods with near neutral or moderately low pH and relatively high aw values (pH ≥ 5.0 and aw ≥ 0.94) and pH 4.4 and aw 0.92 for each factor individually. and absence of *L. monocytogenes* in 25 g or mL in RTE intended for infants or special purposes. Similar regulatory limits for *L. monocytogenes* are adopted worldwide as reported by FAO and WHO [5] According to EFSA Panel on Biological Hazards [6] more than 90% of invasive listeriosis was caused by ingestion of RTE food containing > 2,000 colony forming units (CFU)/g, and that one-third of cases were due to growth in the consumer phase. The authors concluded that awareness should be increased, especially in relation to risk groups and innovative methodologies including monitoring of trends were recommended.

The prevalence of *L. monocytogenes* in ready-to-eat fish products can be influenced by the populations that make up the background microbiota. Competitive-type interactions are expected when this background microbiota is predominantly lactic acid bacteria (LAB), which have the ability to inhibit the growth of *L. monocytogenes* without affecting the sensory characteristics of the product [7], mainly in cold-smoked or smoked foods semi-preserved [8]. LAB are much more used and exploited in dairy, meat and vegetables products compared to fish [9]. Lactic acid bacteria can produce organic acids, hydrogen peroxide, bacteriocins, highly potent small peptides that can inhibit the growth of spoilage and pathogens microorganisms, such as *L. monocytogenes* [10, 11] and thus also prolong the shelf life of the RTE fish product [12]. The use of protective cultures of LAB has been suggested for direct application onto the food surfaces and packaging [13].

Kani-kama is an imitation crab meat developed from surimi which is produced from white fish meat, which is boneless, minced and washed processed in the fish industry into rods [14].The production of kani-kama begins with the preparation of surimi paste from white-meat fish, which consists of a concentrate of myofibrillar proteins obtained from ground fish muscle, washed repeatedly with water, and added with cryoprotectants agents to maintain protein stability during freezing. The operation of washing the fish meat allows the elimination of most of the substances that reduce the stability of the surimi, especially sarcoplasmic proteins that are water soluble, fat, and non-protein nitrogenous compounds. The paste is added of flavouring then extruded, gelatinized, and steamed at 80 to 90 °C for 20 to 40 min. This paste is usually moulded into thin layers, which are rolled, dyed with red dye, transformed into sticks, cut to the appropriate size, vacuum packed in plastic, and finally pasteurized at 65 °C for 30 min and cooled in cold water inside the packaging to be stored frozen [15, 16].

Differently, smoked salmon is a lightly preserved food, which undergoes a process of salting, drying, and smoking. With the application of these steps, it is possible to achieve sensory attributes, modify intrinsic factors of the food, such as the reduction of a_w_, and consequently reduce microbial deterioration and increase the shelf life of the food [17]. However, the pH of cold smoked salmon is in the range of 5.9 to 6.3 and the water activity is from 0.95 to 0.98, not limiting the development as *Listeria* sp of pathogenic microorganisms, such as *L. monocytogenes.* [18, 17].

Bacteria of the genus *Listeria* are ubiquitous microorganisms that are found in a variety of sources such as soil, water, decaying vegetation. For this reason, *Listeria* spp. is an indicator of an inadequate hygiene. and/or cleaning process in food handling areas [19].

Currently, RTE kani-kama are not produced in Brazil. All kani-kama products marketed in Brazil are imported. In the case of smoked and sliced salmon, the raw material is imported. Epidemiological data on the occurrence o*f L. monocytogenes* in RTE fish in Brazil are not known. Meanwhile, reports of the occurrence of *L. monocytogenes* in ready-to-eat foods have intensified in many countries. According to the European Centre for Disease Prevention and Control (EFSA) [20] the occurrence of *L. monocytogenes* varied according to the RTE food category and, and in 2019, ranged from 0.04% for ‘hard cheeses made from pasteurised milk’ up to 4.3% for ‘RTE fishery products with Germany, Italy, Poland and Romania reporting more than 80% of positive samples. In the 2010 to 2012 baseline survey in the European Union (EU) reported by EFSA [21] in which samples were collected from both supermarkets and small shops and a 25-g sample was analysed the prevalence of *L. monocytogenes* in the EU samples was10.4% (9.1 to 11.7%) and 10.3% (9.1 to 11.6%) for packaged smoked or gravid fish. Inoue et al. [22] surveyed retail foods in Japan for the presence and contamination levels of *L. monocytogenes.* The organism was isolated from 5.4% of the 92 smoked salmon samples at MPN values lower than 10/g, and from 3.3% of 213 ready-to-eat raw seafood samples at MPN values from lower than 0.3 to higher than 100/g. They concluded that ready-to-eat raw seafoods are relatively high risk among the foods surveyed.

Giving this reports this research aimed to identify the presence and level of contamination of the pathogen *L. monocytogenes* and of *Listeria* sp. as indicator of the hygiene conditions of RTE kani-kama and smoked and sliced salmon, as well as to know the impact of storing these products under refrigeration after purchasing. In addition to correlating *Listeria* populations with the background microbiota, especially LAB population.

## Materials and methods

### Sampling protocol

Samples of ready to eat fish products, kani-kama and smoked and sliced salmon, were purchased frozen, within the expiration date, from markets in the city of Rio de Janeiro and the metropolitan region between October 2021 and July 2022 and transported in thermal boxes to the laboratory. To simulate the possibility of the consumer storing the product under refrigeration before consuming besides analysing the product immediately, samples were also stored under refrigeration an analysed at some interval times. In each collection, three different brands of products and at least three packages of each were acquired so that a sample of each was analysed initially (time zero) and after different time intervals under refrigeration. Collections were carried out on different occasions totalizing 24 samples of each brand so that different batches of the same brand were sampled.

Frozen, kani-kama and smoked salmon have a shelf life of 2 years stipulated on the packaging. A total of 120 samples were analysed, consisting of 72 samples of kani-kama, from three most popular commercial brands (kani 1, kani 2, kani 3) and 48 samples of smoked and sliced salmon from three commercial brands (SDe1, SDe2 e SD3). Three subunits of the same batch of kani-kama were collected and analysed at three different times: (i) immediately (time 0), (ii) after 7 days and (iii) with 15 days of storage under refrigeration at 5 °C inside untouched vacuum packaging. For smoked and sliced salmon, two subunits of the same batch were collected and analysed at two different times: (i) immediately (time 0), and (ii) after 30 days of storage under refrigeration at 5 °C inside untouched vacuum packaging.

### Reference strains

Food isolates *L. monocytogenes* CLIST 02034 serotype 4ab and CLIST 02045 serotype 4b, *L. innocua* CLIST 02050 *L. ivanovii* CLIST 02057, *L. seeligeri* CLIST 02067, were selected as reference strains for positive control during the development and evaluation of the phenotypic tests as well as PCR and qPCR tests. These strains were obtained from the *Listeria* collection (CLIST) of the Laboratory of Bacterial Zoonoses (LABZOO) of the Oswaldo Cruz Foundation (FIOCRUZ).

### Detection and quantification of *Listeria* sp and *L. monocytogenes* by culturing

The presence of *L. monocytogenes* was detected following the procedure established in ABNT NBR ISO 11290-1 (2020b) [23]. Initially, the enrichment step was performed by weighing 25 g of the sample into 225 ml of the primary enrichment broth, demi-Fraser (Himedia, Mumbai, India), followed by incubation at 30 °C for 24 h. Subsequently, 0.1 ml of broth was transferred to a tube containing 10 ml of secondary enrichment medium, Fraser broth with incubation at 37 °C for 24 h. This was followed by streaking on the surface of *Listeria* agar plates according to Ottaviani and Agosti (ALOA) (Himedia, Mumbai, India) as well as an additional medium, Palcam agar (Oxoid, Basingstoke, UK), in order to obtain isolated colonies. Plates were incubated at 37 °C for 24 h. All *Listeria* spp. produced turquoise colonies and pathogenic species *L. monocytogenes* and *L. ivanovii* appeared surrounded by a distinct precipitation zone Morphological and biochemical test, such as beta haemolysis, catalase and fermentation of sugars, L-rhamnose and D-xylose were performed to confirm presumptive colonies of *L. monocytogenes*. Results were expressed as presence or absence of *L. monocytogenes* in 25 g.

The enumeration of *Listeria* spp. and *L. monocytogenes* was performed according to the ABNT NBR ISO 11920-2 (2020c) [24], which is the official international reference method for viable cell detection and enumeration, twenty-five grams of each sample were weighed and homogenized with 225 mL of buffered peptone water solution, obtaining a 1:10 dilution. Then, 1.0 ml of this initial dilution was distributed and spread over the surface of five Petri dishes containing ALOA agar. The plates were incubated for 24 h at a temperature of 37 °C. All turquoise colonies surrounded or not, by a distinct precipitation zone, were counted for enumeration of *Listeria* spp. and only those surrounded by the precipitation zone for enumeration of *L. monocytogenes*. To confirm, a pool of five presumptive colonies of *L. monocytogenes* of each type were taken from each Petri dish, for example, with large halo and small halo, carrying out the biochemical tests indicated and described above. For confirmation of the presumptive colonies, PCR/qPCR were additionally used as molecular detection tools for *Listeria* sp. and *L. monocytogenes*, respectively.

### Enumeration of aerobic mesophiles, total psychrotrophs, hydrogen sulphide producers psychrotrophs and lactic acid bacteria (LAB)

The aerobic mesophilic count was performed according to ABNT NBR ISO 4833-2 (2015a) [25]. Twenty-five grams of the sample was weighed in stomacher bags and homogenized in a stomacher homogenizer (Stomacher^®^ 400 lab blender, Seward) with 225 ml of 0.1% peptone water, obtaining a 1:10 dilution. Successive serial decimal dilutions were prepared, and 0.1 mL aliquots of each dilution were seeded in duplicate on the surface of plates containing PCA counting agar (Merck, Darmstadt, Germany) which were incubated at 30 °C for 72 h.

To carry out the total psychrotroph count and of hydrogen sulphide producers psychrotrophs, aliquots of serial decimal dilutions were spread on the surface of Lyngby iron agar containing 20 g/L bacteriological peptone, 3.0 g/L beef extract, 3.0 g/l yeast extract, 0.3 g/l, ferric citrate, 0.3 g/l sodium thiosulphate, 5.0 g/L sodium chloride and 14 g/L agar-agar, with incubation at 10° C for 7 days. Likewise, enumeration of LAB was performed by surface inoculation on plates containing Man Rogosa and Sharpe-MRS agar (Kasvi, Liofilchem, Italy). Incubation was at 30 °C in aerobiosis for 72 h. Results were expressed as colony forming units per gram (CFU/g).

### Detection of *Listeria* spp. and *L. monocytogenes* by genotyping

The preparation of samples for the Polymerase Chain Reaction (PCR) assay consisted of taking an aliquot of ca. 1 mL of the 1:10 dilution of each food sample in buffered peptone water, transferring it to an Eppendorf tube, centrifuging at 2,000 rpm for 2 min, discarding the supernatant and to the pellet was added phosphate buffer (PBS). After homogenizing in vortex (Velp Scientific) it was centrifuged again for another 2 min at 12,000 rpm. Fifty µL of the pellets were transferred to new Eppendorf tubes and kept in a freezer at -20 °C until analysis.

### Genomic DNA extraction protocols

DNA extraction was carried out on pellet, obtained directly from the food samples by the phenol-chloroform extraction method, using ca. 50 mg of sample, 20 µl/mL of proteinase K, 650 µl of buffer solution (10 mM Tris-HCl pH 8.0; 10 mM EDTA pH 8.0; 250 µl/mL of PBS), which was homogenized in a Bead Beater (L-Beader 6 Loccus) for 60s at 3.500 rpm and incubated at 56 °C for 1 h with agitation at 650 rpm. Next, 920 µL of phenol pH 8.0 was added, gently vortexed and centrifuged at 14,559 rpm for 10 min, and the supernatant was transferred to a new tube containing 500 µL of chloroform. After another vortex agitation and centrifugation at 14,559 rpm for 10 min, the supernatant obtained was transferred to another tube containing 50 µL of 3 M sodium acetate pH 5.2. Then, 500 µL of 100% ethanol was added, vortexed and precipitated by freezing in a -20 °C freezer for 1.5 h. After this time, the DNA was recovered by centrifuging for 10 min at 14,559 rpm, discarding the supernatant, and adding 500 µL of 70% ethanol to the pellet. The tube was centrifuged for 10 min at the same speed described above, and the excess ethanol in the pellet was evaporated for approximately 30 min. The DNA was then eluted in 50 µL of elution buffer and quantified using a Nano Drop (2000 Termo scientific) and used as a template for PCR and qPCR.

DNA extraction from presumptive colonies of *Listeria* sp. and *L. monocytogenes*, were performed according to the following protocol. Initially, the isolated colonies were transferred to tryptone soy broth and incubated at 36 °C for 22 to 24 h. Next, 1 mL of the broth was transferred to 1.5 mL Eppendof tubes, centrifuged at 10,500 rpm for 10 min and the supernatant was discarded. The pellet was added of 1 ml of PBS, vortexed and centrifuged again and the supernatant was discarded. After performing this process twice, the pellets were stored in a freezer at -20 °C until the moment of analysis. DNA extraction was performed using a commercial DNeasy^®^ Blood & Tissue Kit (250) following the manufacturer’s recommendations. After extraction, the samples were quantified in a spectrophotometer (Nanodrop ND-2000^®^, Thermo Scientific, Wilmington, DE, USA) and standardized at 50 ng/µL. Samples were kept in a -20 °C freezer until molecular analysis.

### Detection of *Listeria* spp

For detection of the genus *Listeria*, oligonucleotides, S2F (5’-GCCTACAAGTAGTTAGAGCC-3’) and S2R (5’-ACTGGTACAGGAATCTCTAC-3’) were used, according to Paillard et al. [26] with modifications. The final volume of each PCR reaction was 25 µL, and contained 1X of Platinum Taq Buffer 50 mM KCl, 10 mM Tris-HCl (pH 8.3), 2.0 mM MgCl2, 0.4 mM dNTP, 0.6 mM primer 1.0 U Taq polymerase, 0.3 mM of DNA from samples and controls. Amplification was performed in a thermocycler (ProFlex, Applied Biosystems) with a temperature profile for initial denaturation set at 94 °C for 5 min, followed by 40 cycles, each comprising a denaturation phase at 94 °C for 1 min, an annealing at 50 °C for 1 min and an extension step at 72 °C for 1 min, followed by a final extension step at 72 °C for 7 min. After amplification, electrophoresis was performed on a 2.0% agarose gel stained with ethidium bromide and visualized on a UV transilluminator (Life Technologies).

### Detection of *L. monocytogenes*

For the detection of the specie *L. monocytogenes* the following primers were used, IGS1(5’-GGCCTATAGCTCAGCTGGTTA-3’) and IGS2 (5’GCTGAGGCTAAGGCCCCGTAAA-3’) according to the methodology described by Rantsiou et al. [27] with modifications. The qPCR reactions with a volume of 12 µL, containing: 3 µM of DNA, 1X of SYBR^®^ Green PCR Master Mix, 0.4 mM of each primer, were performed using the StepOnePlus equipment (Applied Biosystems^®^, ThermoFisher Scientific, Wilmington, DE, USA) with the following thermocycling conditions: initial denaturation at 95 °C for 10 min, followed by 50 cycles of 95 °C for 30s, 54 °C for 30s, and 72 °C for 30s.

### PCR analytical sensitivity

The analytical sensitivity of PCR was evaluated using suspensions containing decreasing amounts of cells (10^6^ to 10 CFU/mL) of total DNA extracted from the strains of *L. monocytogenes* CLIST 02034 and CLIST 02045. PCR was run to determine the number of copies in each dilution point and the estimated calculation was performed according to the equation. Number of copies equals to 6.02 × 10^23^ (copies per mol) x DNA concentration (g)/ target size*(*base pair) x 660 (g/mol/bp), using the amount of the total genome of *L. monocytogenes* for its application.

The number of copies ranged from 1 to 1 × 10^6^ per µL, with seven separate dilution series performed for each point on the curve.

For qPCR, the number of copies evaluated varied using suspensions in decreasing amounts of cells from 10^6^ to 10 per µL and each curve point was performed in triplicate. Linear regression along with the coefficient of determination (R^2^) were employed to evaluate the optimization of the qPCR assay.

### Statistical analysis

All data are presented as means ± standard error of the mean (SEM). GraphPad Prism 8 statistical software (La Jolla, CA, USA) was used for statistical analyses. Rout’s test was used for detecting outliers. The assumption of normal data distribution was assessed with the Shapiro-Wilk test. Data that passed the normality assumption were analyzed using parametric methods. In this case, between-group comparisons were analyzed by two-way ANOVA. When applicable, Tukey post hoc tests were performed, and significance was determined using p-values. Differences were considered statistically significant when *p* ≤ 0.05.

## Results and discussion

In the present study, detection of *Listeria* sp was carried out in two ways, from presumptive *Listeria* colonies grown in ALOA culture medium as well by PCR directly from the food matrices, kani-kama and smoked and sliced salmon. Detection of *L. monocytogenes* was performed only by qPCR, directly from the food matrices.

The band corresponding to the *Listeria* sp. gene has 890 base pairs (Figure S1) The PCR detection limit was 10 target copies containing the *L. monocytogenes* gene (Figure S2). The detection limit of *L. monocytogenes* using the qPCR method was 100 copies. The qPCR method was used only for qualitative detection The coefficient of determination was 0.991, which is very close to the optimal theoretical ideal of 1.0. The efficiency of the reaction was 114,11%. The number of quantification cycles (Qc) varied from 20.68 cycles in the first suspension (10^6^ CFU/mL) to 32.15 cycles in the last suspension (10^2^ CFU/ml). In real-time PCR, the amount of DNA is measured after each cycle via fluorescent dyes that yield increasing fluorescent signal in direct proportion to the number of PCR product molecules (amplicons) generated. Despite it was expected greater sensitivity the qPCR technique, detection limit was higher than that observed with PCR which detected up to 10 copies of the genus *Listeria* target gene. This was probably due to the efficiency of the primer used. The dissociation curve of the *L. monocytogenes* fragment amplified by the Polymerase Chain Reaction in real time, using the strain *L. monocytogenes* CLIST 02045 as a positive control, showed a melting temperature (Mt) of approximately 81 °C without the presence of a dimerization peak.

### Detection of *Listeria* sp. positive samples among manufacturers of ready-to-eat (RTE) fish products

**Fig. 1:**
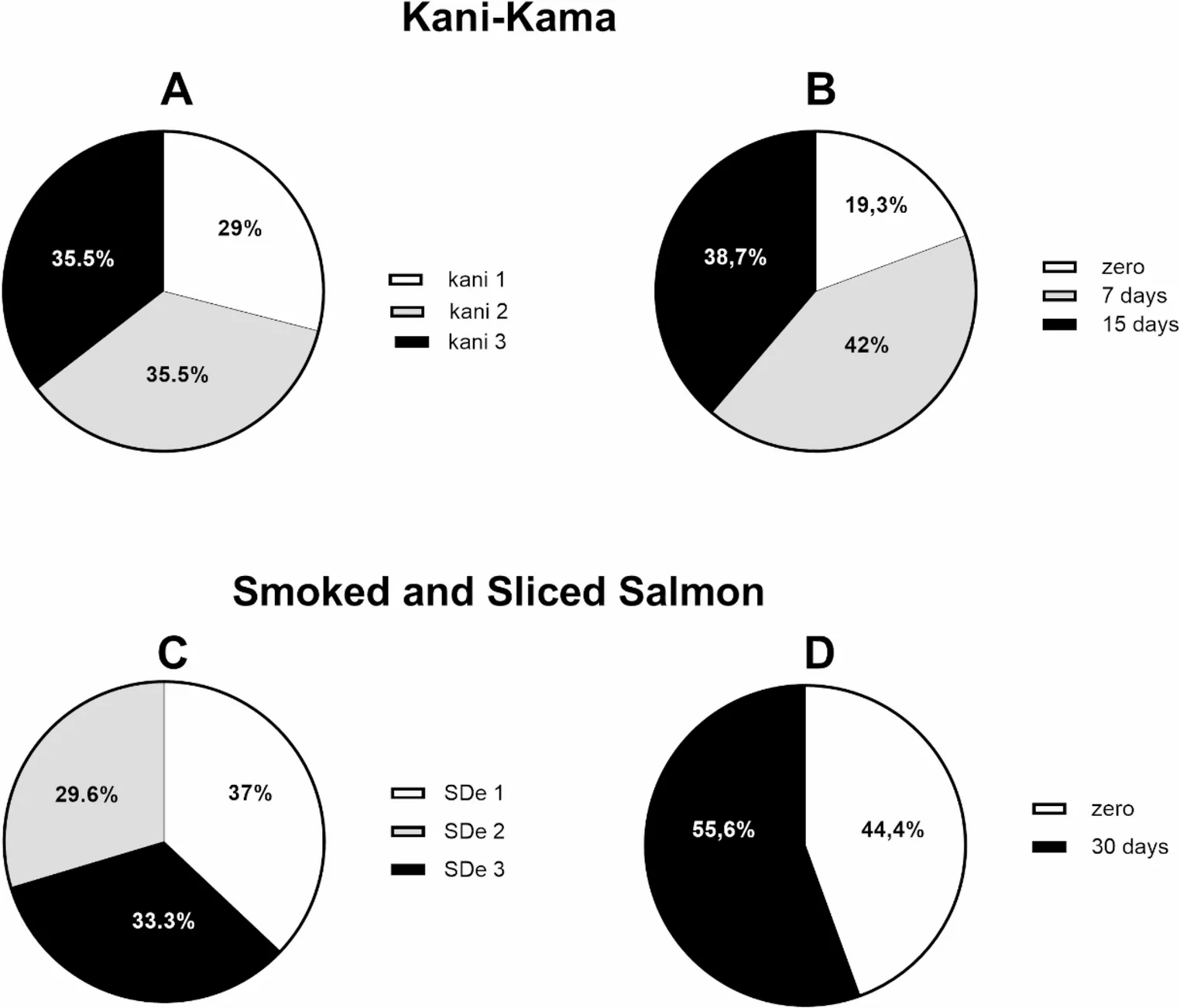
Percentage of kani-kama and smoked and sliced salmon samples testing positive for *Listeria* sp. detected by PCR directly in the food matrices, among different commercial brands (**A** and **C**) and at sampling times of t zero, t 7 days and t 15 days for kani-kama and t zero and t 30 days for smoked salmon under refrigeration at 5 °C (**B** and **D**)

*Listeria* sp. is an indicator of an inadequate hygiene. and/or cleaning process in food handling areas [19] due to its ubiquity in the environment. The number of kani-kama samples contaminated with *Listeria* spp increased after 7 days of refrigerated storage, but contrary to the expectations, did not increase from the 7th to the 15th day, indicating that the initial contamination of each package may not be exactly the same. The percentage of *Listeria* sp. positive samples in the three commercial brands of kani-kama are shown in Fig. 1-A. The number of contaminated samples among the three manufacturers did not differ significantly (*P* > 0.05) leading to the conclusion that the hygienic manufacturing practices employed by the different manufacturers are very similar. When analysed at different times, the smallest number of positive samples was detected immediately after acquisition (time 0), increasing afterwards with refrigerated storage (Fig. 1-B).

The percentage of positive smoked and sliced salmon samples for *Listeria* sp. was similar among the three manufacturers, ranging from 29.6% to 37%. (Fig. 1-C). The occurrence of positive samples was higher within 30 days at 5 °C, as it was expected. *Listeria* spp. are capable of growing under refrigeration, reaching detectable levels (Fig. 1-D). It is concluded that the cold chain must be applied correctly as described on the packaging of ready-to-eat fish products to avoid an even greater risk to product safety.

### Detection of *Listeria* sp. and *Listeria monocytogenes*

**Fig. 2:**
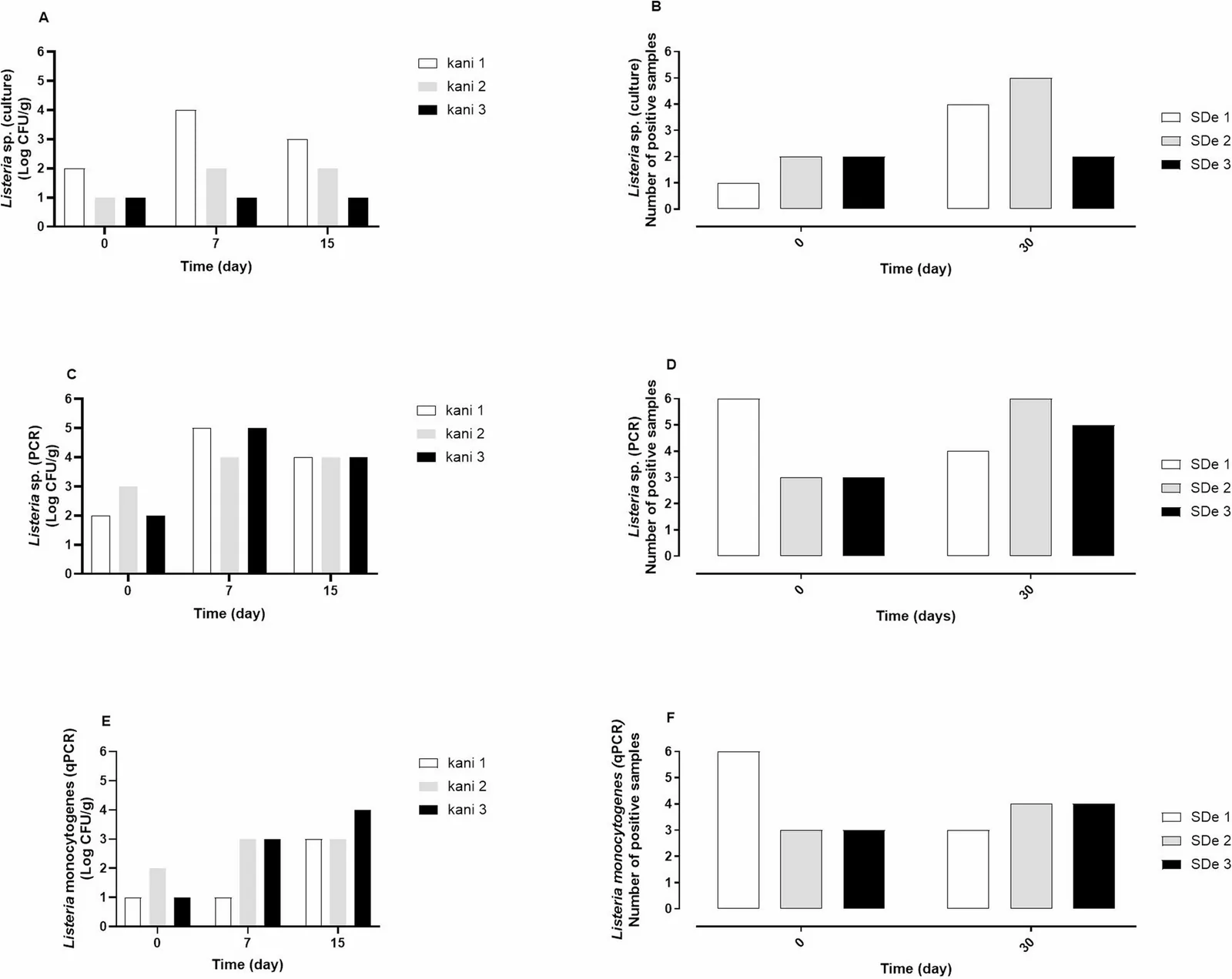
Presence of *Listeria* sp.in three different brands of kani-kama and smoked and sliced salmon samples detected by the traditional culture method on ALOA agar (**A** and **B**) and by PCR directly from the food matrices (**C** and **D**). Presence of *Listeria monocytogenes* in three different brands of kani-kama and smoked and sliced salmon detected by qPCR directly from the food matrices (**E** and **F**). Kani-kama was analysed immediately (zero time) and after 7 days and 15 days of storage at 5 °C whereas smoked and sliced salmon was analysed immediately (zero time) and after 30 days of storage at 5 °C

All brands of kani-kama or smoked salmon showed at least one sample contaminated with *Listeria* sp. when examined immediately after purchase (time 0). (Fig. 2) Out of 72 kani-kama samples, 17 (23,6%) tested positive for *Listeria* sp. by the traditional culture method (Fig. 2-A), while 33 (45,8%) were positive by PCR (Fig. 2-C). Sixteen out of 48 samples (33,3%) of smoked salmon were detected positive for *Listeria* sp, by culture method (Fig. 2-B) while 27 (56.2%) were positive when the samples were analysed by PCR (Fig. 2 -D). According to the results of culture method, the highest number of positive smoked salmon samples was observed with the brand SDe 2 at the 30th day under refrigeration (Fig. 2-B). Surprisingly, the brand SDe 1, which initially (time 0) showed only one positive sample by the culture method, when analysed by PCR the number of samples detected positive increased to six, being greater than the number of positive samples after 30 days at 5 °C (Fig. 2-D).

The PCR technique proved to be more sensitive than the traditional culture method. The PCR method showed a higher level of detection, with sensitivity to detect the target gene even at a low level of contamination in the sample. Official culture methods are laborious and time-consuming [28]. In addition, it is possible to underestimate the number of microorganisms due to a viable but non-cultivable state (VBNC) [29], which would explain the higher level of detection by PCR.

Some manufacturers establish a 12-month shelf life for kani-kama stored frozen at -18 °C, and the products were purchased over a period of nearly a year, already containing *Listeria* sp. at levels detectable by culture and PCR methods.

This high number of positive samples was unexpected for kani-kama, given that it is a thermally processed product. Mazzotta [30] stated that the possibility of kani-kama contamination after the cooking steps and after packaging is very low, and pasteurization post vacuum-packaging is an adequate way to prevent *L. monocytogenes* contamination. McDermott et al. [31] undertake a thermal investigation to provide thermal inactivation data for *L. monocytogenes* in crab meat. They concluded that current pasteurization conditions (70 °C for 2 min) would achieve complete destruction of any *L. monocytogenes* present in crab meat. In the case of kani-kama, which is an emulsified fish food produced from white fish meat, pasteurization occurs inside vacuum packaging at 65 °C for 30 min [16]. Nevertheless, in this research several samples were contaminated with *L. monocytogenes*. Kani-kama is not crab meat but an imitation of crab meat made of white fish meat forming an emulsion containing added solids that can protect bacteria from heat. Besides, the raw material can be an important source of contamination. Recontamination after heat treatment is not likely to occur since the product is pasteurized inside the vacuum package.

Applying the qPCR technique to detect the specie *L. monocytogenes*, 21 samples of kani-kama out of 72 (29.2%) were positive, with 4 positive samples already at the time of purchasing (time 0), 7 after 7 days and 10 within 15 days storage at 5 °C (Fig. 2E).

It was found that among all samples from the three brands, at least one sample tested positive for the pathogen at each of the different times evaluated. The high prevalence of the pathogen in RTE fish products which will not undergo additional heating before consumption highlight a risk for the consumer.

Kani brand 1 showed an increase in the number of samples contaminated after 15 days of storage, while the number of samples contaminated with *L. monocytogenes* observed for the other two brands was already hight at the 7th day of refrigerated storage (Fig. 2E).

According to Cabedo et al. [32] Farber & Peterkin [33] and González et al. [34] the incidence of L. *monocytogenes* in crab analogues (kani-kama) is low, ensuring that this is a ready-to-eat food of high quality from the hygienic and sanitary point of view. However, the results found in the present study reveal possible failures in the process, either in the manufacturing or in the cold chain, despite the kani-kama being pasteurised inside the vacuum packaging. One factor that was observed and that may have contributed to the high occurrence of *L. monocytogenes* in kani-kama was the temperature at which the samples were found in supermarkets. Some of them were defrosted, indicating that supermarkets in the outskirts of Rio de Janeiro, where the product was purchased, which may favour the growth of psychrotrophic microorganisms such as *L. monocytogenes* [35, 36].

In 2018, a multistate outbreak of food borne infections linked to crabmeat that resulted in 26 ill people was investigated in the U.S.A. State and U.S. Food and Drug Administration (FDA) laboratories recovered *V. parahaemolyticus*, *Salmonella* spp., and *L. monocytogenes* isolates from crabmeat samples collected from various points of distribution and conducted phylogenetic analysis of whole-genome sequencing data to determine the source of crabmeat [37]. 

It is important to emphasise that only one of the brands warned on the label that all the package content should be consumed soon after thawing. Frequent temperature variations in ready-to-eat fish products can also impair the sensory characteristics by favouring the growth of spoilage microorganisms that produce undesirable odours making the product unacceptable for consumption [38, 39]. An outbreak involving two healthy adult people in Canada was linked to consumption of a crab analogue (kani-kama) containing a high number of *L. monocytogenes* cells, 10^6^ CFU/g. It was suggested that kani-kama may have been stored for a long time at temperatures above 10 °C, which may have favoured the rapid growth of *L. monocytogenes*. To prevent the spread of foodborne illnesses involving ready-to-eat products, temperature control during storage is critical to prevent or inhibit the growth of this pathogen [40].

Out of 48 samples of smoked and sliced salmon 23 samples of smoked and sliced salmon (47.9%) were positive for *L. monocytogenes*, 12 of which already by the time of purchasing (time 0) and 11 after 30 days of storage at 5 °C (Fig. 2F), denoting a high prevalence of *L. monocytogenes* in the product, even under freezing. Samples of smoked and sliced salmon were acquired in markets in the cities of Rio de Janeiro and Niteroi, where the products were properly frozen at the moment they were bought. Cold-smoked salmon is a food that presents a high risk of contamination by *L. monocytogenes*, as several factors contribute, especially the smoking stage, as well as inadequate storage temperature, and for these reasons it has been the subject of many studies in recent years. In addition, it is ready-to-eat food and does not undergo heating to ensure the elimination of pathogenic microorganisms that may be present [41, 42, 43].

### Enumeration of *Listeria* sp. and *L. monocytogenes*

Enumeration of presumptive colonies of *Listeria* sp. and *L. monocytogenes* was performed on ALOA agar and was confirmed for *Listeria* sp. and *L. monocytogenes* by PCR and qPCR techniques, respectively. This hybrid methodology that combines cultivation on ALOA agar with confirmation of suspected colonies through genotyping was adopted to try to overcome the limitations of each method. The main drawback of molecular methods is that it detects even dead cells. On the other hand, it was observed that traditional phenotypic methods such as assessing beta hemolysis, catalase activity, and fermentation of L-rhamnose and D-xylose to confirm presumptive colonies on ALOA agar are not sufficient on their own to differentiate *L. monocytogenes* from other species. To avoid the laborious work with supplementary biochemical tests, genotypic confirmation was chosen.

**Fig. 3:**
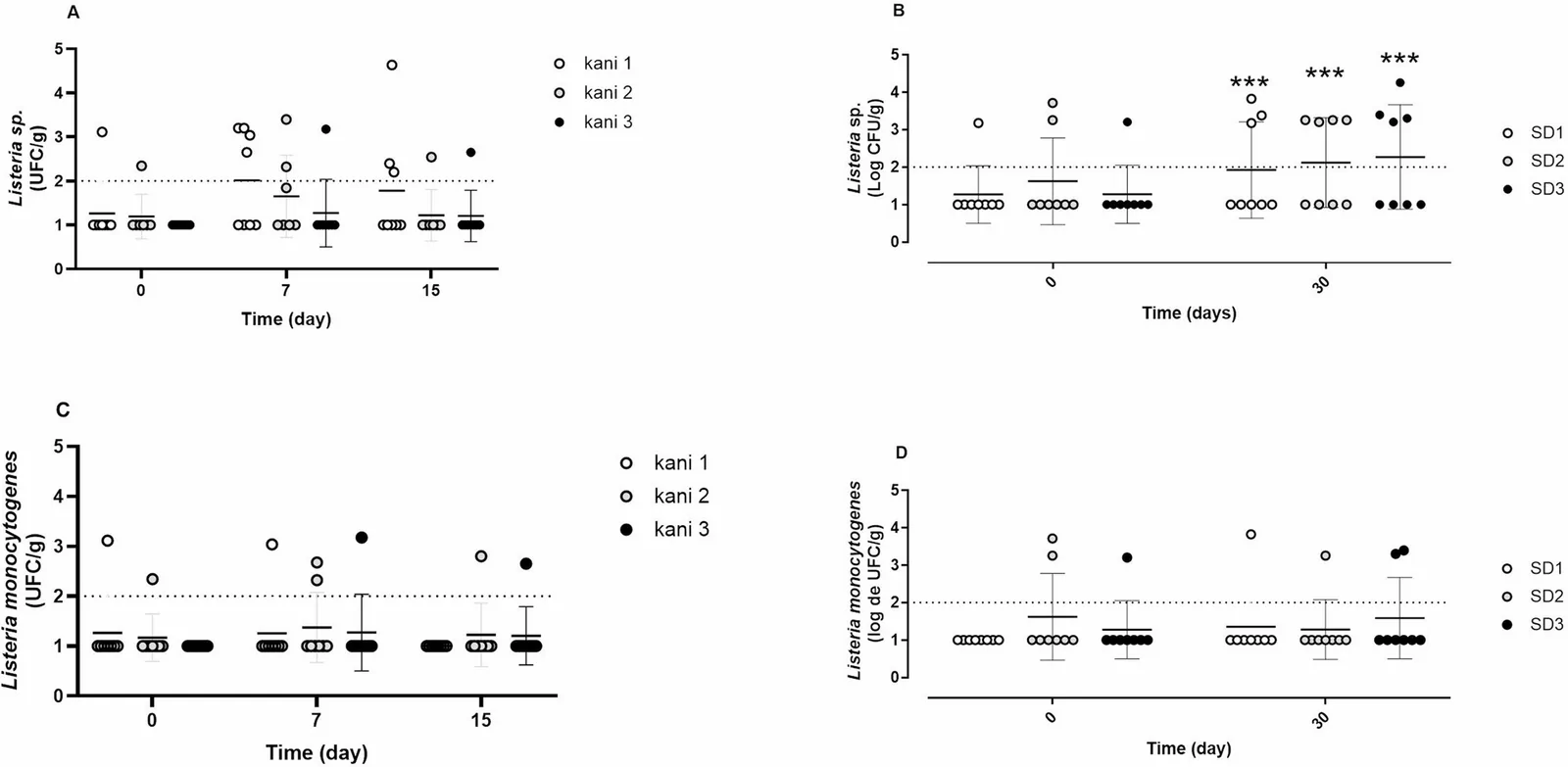
*Listeria* sp. (**A** and **B**) and *Listeria monocytogenes* (**C** and **D**) counts in samples from three commercial brands of kani-kama (**A** and **C**) on the day of acquisition (time 0) and after 7 days and 15 days of storage at 5 °C and of smoked and sliced salmon (**B** and **D**) on the day of acquisition (time 0) and after 30 days of storage at 5 °C. Counts were performed on ALOA agar and confirmed by PCR and qPCR for *Listeria* sp and *L. moocytogenes*, respectively. For calculation purposes, counts < 1 Log CFU/g were considered 1. The dotted line in corresponds to the limit established in microbiological standards for foods regarding *L. monocytogenes* [4}. *** represents a significant difference by Tukey’s test (*p* < 0.001)

All three brands of kani-kama showed quantifiable levels of the microorganism even as soon as it had been purchased. The kani brand 1 within 7 days of storage at 5 °C showed the highest mean count of *Listeria* sp, 2,0 ± 1.1 log CFU/g (Fig. 3-A). The average counts of *L. monocytogenes* did not exceed the maximum limit of 2 Log CFU/g). However, 11 individual samples presented counts of more than 2.0 Log CFU/g up to 3,2 Log CFU/g (Fig. 3-B), which could represent a health risk, especially for consumers with low immunity.

For healthy consumers, it is scientifically recognised that only ingestion of food containing concentration of *L. monocytogenes* over the limit of 100 cfu/g is potentially injurious to health [6]. However, infants and consumers with weakened immune defences are highly susceptible to *Listeria monocytogenes* and cannot be exposed to food containing this pathogen at any concentration. Regulatory limits for *L. monocytogenes* recommended by Codex Alimentarius [5] are adopted in Brazil and in most countries The European Commission Regulation No 2073/2005 [44] indicated that *L. monocytogenes* not detected in 25 g” should apply to all situations where those foods are placed on the market during their shelf-life and for which the producing food business operator has not been able to demonstrate, to the satisfaction to the competent authority, that the level of *L. monocytogenes* will not exceed the limit of 100 cfu/g during their shelf-life.

While MAPA, the Brazilian Ministry of Agriculture, Livestock, and Supply [45] and ANVISA the Brazilian Health Regulatory Agency [4] have standards and programs for monitoring *L. monocytogenes* in foods such as RTE foods, there is no official epidemiological data on outbreaks of *L. monocytogenes*, since this pathogen is not subject to mandatory notification to health authorities when detected in clinical samples.

**Fig. 4:**
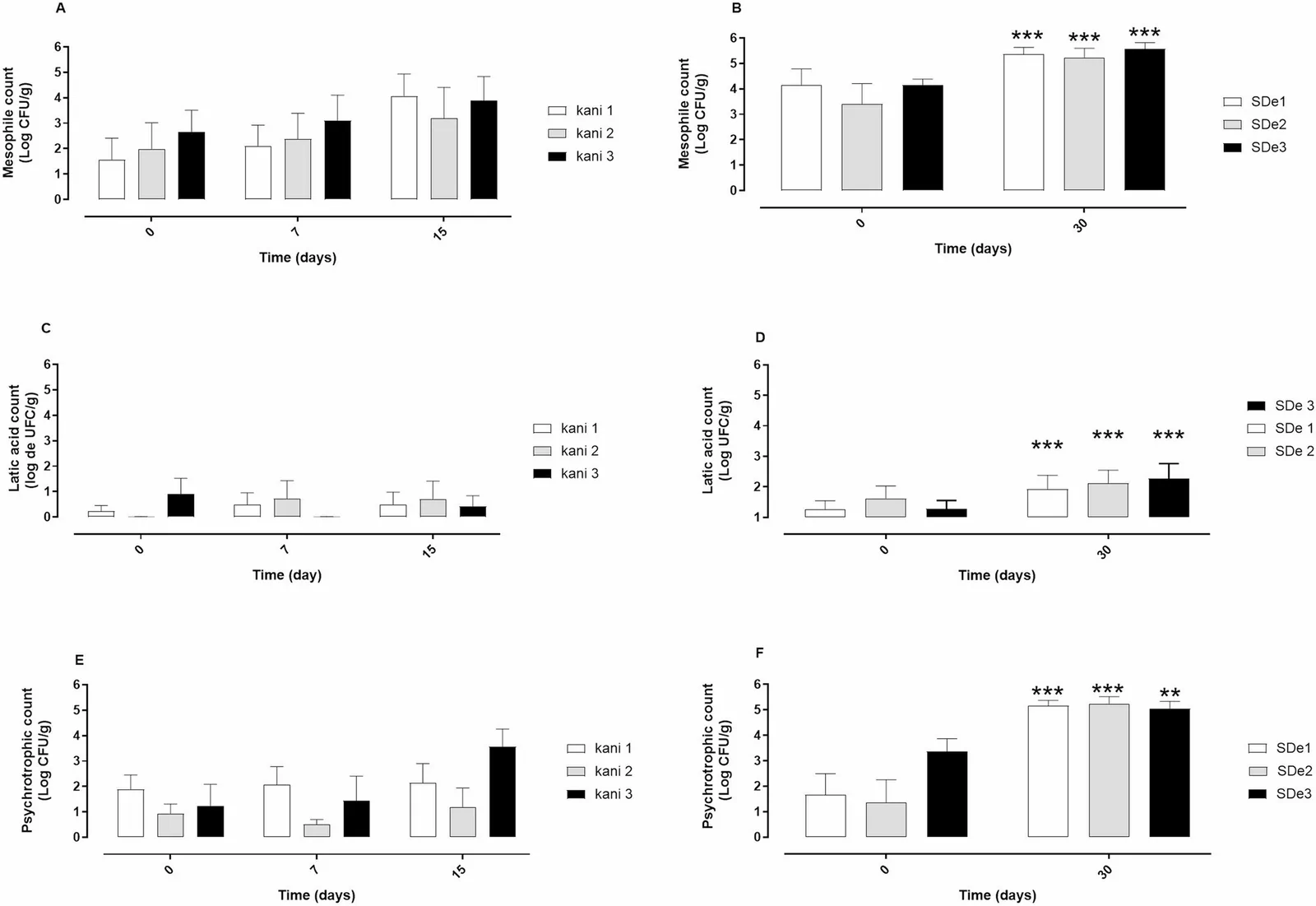
Mean counts and standard deviation of total aerobic mesophilic (**A** and **B**), lactic acid bacteria (**C** and **D**) and psychrotrophs (**E** and **F**) counts in samples of kani-kama (**A**, **C**, **E**) and of smoked and sliced salmon (**B**, **D**, **F**) from three commercial brands, analysed immediately (zero time) and during refrigerated storage at 5 °C for 7 and 15 days for kani-kama and for 30 days for sliced smoked salmon. *** represents a significant difference by Tukey’s test (*p* < 0.001) and ** represents a significant difference by Tukey’s test (*p* < 0.01)

The results of smoked salmon counts of *Listeria* sp can be seen in Fig. 3-C A significantly higher number of *Listeria* sp. after 30 days of storage (*P* < 0.05) was observed, confirming that *Listeria* sp. is able to grow under refrigeration. Despite that only SDe2 and SDe3 salmon brands average counts exceeded the count of 2 log CFU/g, individually, samples with counts greater than 2 Log CFU/g were detected in all the three brands evaluated. The number of samples with counts of *Listeria* sp greater than 2 Log CFU/g increased from four in the day of purchasing (time 0) to 11 after 30 days of refrigerated storage (Fig. 4-C).

*L. monocytogenes* average counts in smoked salmon were below 2 log CFU/g both initially (time 0) and after 30 days under refrigeration. while the number of individual samples that exceed this count went from three units at time 0 to four after refrigerated storage (Fig. 3-D). Heir et al. [46] evaluated a challenge test with cold smoked salmon with reduced sodium content at different times 0, 7, 12, 19 and 29, and found that after 6 days of storage at 4 °C the count of *L. monocytogenes* was 3 log CFU/g, which was considered low by the authors. Within 29 days of storage at the same temperature did not exceed 6 log CFU/g. The authors concluded that growth of *L. monocytogenes* in cold smoked salmon was generally independent of the type of mineral salt but emphasize the importance of keeping a continuous cold chain from production through storage. During the salmon smoking and slicing process, some equipment is used, such as salting injectors, a slicer and a vacuum sealer. Slicing and vacuum packaging can be considered two critical control points, because if salmon contamination occurs during these steps, it will not be possible to eliminate pathogenic microorganisms in subsequent steps, as no additional temperature treatment is applied at any stage of the process [47] Consequently, there must be a very strict quality control throughout the process, especially between one stage and another [48, 49].

The presence of *L. monocytogenes* in ready-to-eat foods, even heat-treated, indicates a failure in the process or post-processing that can occur due to cross-contamination through surfaces in direct contact with food, equipment, or food handlers [32]. Fleming et al. [50] described an outbreak of listeriosis involving pasteurized milk and raised questions about the ability of pasteurization to eradicate a large inoculum of *L. monocytogenes* from contaminated raw milk. Inspections at the plant revealed no evidence of improper pasteurization, but multiple serotypes of *L. monocytogenes* were isolated from raw milk obtained from these farms after the outbreak. In the present work, somehow, the heat treatment employed was insufficient to completely eliminate completely eliminate *Listeria monocytogenes* in kani-kama.

The equipment cleaning and disinfection involved in the processing must be carried out with effective sanitizers to reduce or eliminate biofilms of *L. monocytogenes* [51]. The application of sanitizers must be carried out frequently, mainly between one stage and another to avoid any contamination during or after the process [47]. As *L. monocytogenes* is an opportunistic bacterium and can easily grow in processing facilities, drains and walls, it may compromise the integrity of the final product. Corrective actions such as the implementation of an environmental monitoring program is a tool that would significantly improve the control of contamination with the pathogen [52]. Environmental monitoring programs are essential to prevent *L. monocytogenes* contamination [5]. as it is widespread in produce-growing environments, mainly in soil, but also in water, decaying vegetation, moist environments and wildlife. Gill et al. [53] monitored environmental sites of cutting vegetables, cutting fruits and preparing salads. Among facilities, prepared salad showed the greatest *L. monocytogenes* prevalence of 37%. Yoon et al. [54] evaluated the antimicrobial effects of different levels of a potassium lactate plus sodium diacetate against the growth and survival of *L. monocytogenes* and concluded that addition of these antimicrobial substances at all tested levels decreased the surviving populations of this pathogen.

### Counts of aerobic mesophilic microorganisms, lactic acid bacteria (LAB), and psychrotrophic microorganisms in kani-kama and sliced smoked salmon

It was found that the number of aerobic mesophiles in kani-kama, even after 15 days of refrigerated storage at 5 °C, did not exceed 4 log CFU/g (Fig. 4-A), showing no difference between storage times as the standard deviation between samples was hight. The process for obtaining kani-kama is relatively extensive and requires strict quality control, especially in the surimi processing steps, but the fact that the product is pasteurized inside the package contributes to the low counts [55]. Aerobic mesophilic counts in the smoked and sliced salmon samples are shown in (Fig. 4-B). Significant differences (*P* < 0.001) were observed between storage times under refrigeration at 5 °C. In none of the samples the count of aerobic mesophilic exceeded 6 log CFU/g. However, even so, changes in appearance, odour and texture were observed, with noticeable softening of the product. These characteristics indicate that cold smoked salmon remains a highly perishable product and that some improvements are still needed in relation to good manufacturing practices at all stages of manufacturing and distribution.

LAB population in kani-kama was very low even after storage at 5 °C (Fig. 4-A). The low number of lactic acid bacteria in kani-kama did not allow any conclusion about interactions with other microbial groups present, including *Listeria* spp. It is concluded that due to the pasteurization step that the kani-kama rods are subjected to within the vacuum packaging itself, it leads to the elimination of a large part of this microbiota.

In the smoked and sliced salmon, lactic acid bacteria showed significant growth (*P* < 0.05) within 30 days under refrigerated storage compared to the moment of purchasing it frozen (time time 0) (Fig. 4-D) but the count of none of the samples exceeded 4 log CFU/g, with average counts of 2 log UFC/g. It was also not possible to establish any type of interaction between the population of *L. monocytogenes* and of lactic bacteria.

In recent years, several studies with lactic acid bacteria have been developed with the aim of biopreserving fish products, especially ready-to-eat products, proving to be a very promising natural preservation method. It consists of inoculating selected microorganisms in food products, mainly lactic acid bacteria with the potential to inhibit the growth of pathogenic microorganisms such as *L. monocytogenes* and may also increase the shelf life of the product. They are therefore indicated for biopreservation due to their natural antibacterial properties, even when they are part of the natural microbiota of ready-to-eat foods [36–56, 57].

In the study developed by Leroi et al. [58] and Rorvik et al. [59] with cold smoked salmon vacuum packed and kept refrigerated at 4 °C, a shelf life of 3 to 5 weeks was observed, in relation to sensory criteria, therefore a durability of 28 days at 5 °C. A mixture of cultures of lactic acid bacteria and Gram-negative bacteria were identified as the main microorganisms of the background microbiota.

Biopreservation with deliberate inoculation of strains capable of competing with *Listeria*, possible, could represent a safer way to consume smoked salmon, even if kept refrigerated. Although higher in smoked salmon kept refrigerated than in kani-kama, the maximum average LAB count did not exceed 3 Log CFU/g and did not significantly influence *L. monocytogenes* population. It is concluded that numbers of lactic acid bacteria detected in smoked and sliced salmon samples are too low to exert negative or positive interaction with *L. monocytogenes*.

The contamination with psychrotrophic microorganisms in the kani-kama samples was low (Fig. 4-E), possibly because most of these microorganisms are sensitive to the heat treatments applied in the processing of kani-kama. The highest growth was observed with the kani 3 brand at 15 days of refrigerated storage. However, the average count did not exceed 4 log CFU/g, considered a microbial load satisfactory so that the product meets minimum hygienic requirements.

**Fig. 5:**
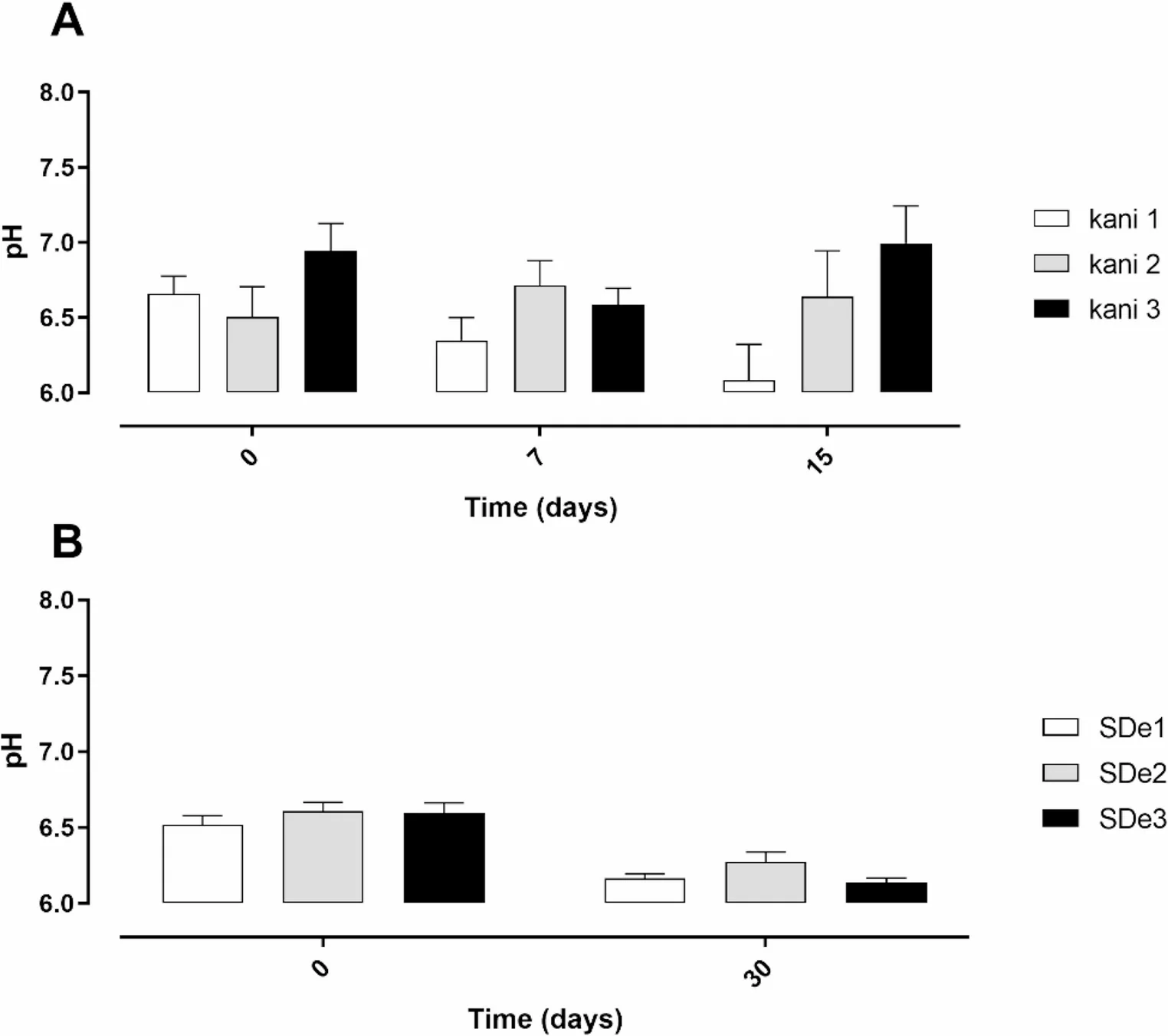
pH averages of three different commercial brands of kani-kama (**A**) and sliced smoked salmon (**B**) analysed immediately (zero time) and during refrigerated storage at 5 °C for 7 and 15 days for kani-kama (**A**) and for 30 days for sliced smoked salmon (**B**)

Population of psychrotrophic microorganisms in the samples of smoked and sliced salmon increased significantly (*P* < 0,001) during the 30 days of storage at 5 °C (Fig. 4-F), in a similar way to what was observed in the population of mesophilic aerobes. After 30 days under 5 °C refrigeration, despite the counts not exceeding 6 log CFU/g, all samples already had unpleasant odour. Nevertheless, hydrogen sulphide-producing psychotropic microorganisms were detected in only two samples. Furthermore, the pH of smoked salmon, after 30 days under refrigeration, was reduced, indicating that the microbiota present was not predominantly proteolytic (Fig. 5).

Seafood main spoilage microorganisms are aerobes, such as *Pseudomonas*, and facultative anaerobes, such as *Shewanella*. However, when the product is vacuum packed, as is the case of kani-kama and cold smoked salmon, facultative anaerobic and anaerobic microorganisms become predominant. This processing then does favour the growth of trimethylamine oxide reducers, such as *Shewanella putrefaciens* which also produces H_2_S and *Photobacterium phophorium*, the latter from temperate waters that is tolerant to increased CO_2_ concentration in modified atmosphere packaged products. Both are psychrotrophic and produce malodorous compounds, significantly reducing the quality of the fish and mainly reducing the shelf life of the product [60].

The main microorganisms in the background microbiota of cold-smoked and vacuum-packed salmon are *Photobacterium* followed by *Carnobacterium* and other lactic acid bacteria, *Enterobacteriaceae* and marine vibrios, *Brochothrix* spp. [46–58–61]. In this sense, according to Gambarin et al. [62] who developed a study with RTE fish products and identified that the psychrotrophic load was high in samples positive for *L. monocytogenes*. However, in the present study, no interaction was found between the presence of *L. monocytogenes* and the load of mesophiles or psychrotrophs, both in kani-kama and in smoked salmon.

In the study carried out by Cardinal et al. [63] with cold smoked salmon, it was identified that 21% of the 57 cold smoked salmon products had a high level of trimethylamine oxide reduction producers, after two weeks of storage at 4 °C corroborating with the off odours observed in the food. These authors also point out that ready-to-eat fish remains a highly perishable product and that improvements are still needed in relation to hygienic conditions during product processing. Although kani-kama or smoked and sliced salmon showed average numbers of *L. monocytogenes* less than 2 Log CFU/g, 11 units of kani-kama (15%) and 7 of smoked and sliced salmon (14.6%) showed numbers greater than 2 log CFU/g. A greater number of positive samples was observed during refrigerated storage compared to recently purchased frozen samples, indicating that refrigeration for a long time can pose an additional health risk, even for heat-processed products like kani-kama.

## Conclusions

*L. monocytogenes* average counts remained within the limit of up to 1 × 10^2^ CFU/g. However, one individual count of each commercial brand sample of kani-kama and of smoked salmon, showed higher *L. monocytogenes* counts of up to 1,6 × 10^3^ CFU/g, some even immediately after purchasing, indicating inadequate hygiene and/or cleaning processes in food-handling areas. It is concluded that somehow, the heat treatment employed was not enough to completely eliminate *L. monocytogenes* even from thermal-processed foods like kani-kama. These RTE food products should remain properly frozen in markets and after purchase until thawing/opening. They should also not be kept under refrigeration for prolonged periods after defrosting, as keeping it refrigerated for a long time can lead to an increase in the population of *L. monocytogenes* to potentially dangerous levels.

Unlike kani-kama, which is a heat-processed product, a higher background microbiota count was detected in smoked salmon. However, this background microbiota apparently did not influence the growth of *Listeria* spp.

## Supplementary Information

Below is the link to the electronic supplementary material.

Supplementary Material 1
