# Supplementary material for: How safe from Listeria monocytogenes are frozen ready-to-eat fish products, either cooked or smoked, when stored under refrigeration after purchasing?
Source: Braz J Microbiol. 2026 Oct 5;57(1):283. doi: 10.1007/s42770-026-02103-7 (PMC13638846; doi:10.1007/s42770-026-02103-7)
Supplement: Supplementary file 1 [file 42770_2026_2103_MOESM1_ESM.docx]

**Supplementary material**

**L	10^6^	10^5^	10^4^	10^3^	10^2^	10^1^**

Figure S1: Detection limit of *Listeria* sp. The suspension containing 10^6^ CFU/mL corresponds to the highest quantity of target gene and 10 CFU/mL to the lowest number of cells detectable by the target gene. Well L corresponds to the 100bp molecular weight marker (Invitrogen®).

pb 890>

Figure S2: Electrophoresis gel for PCR assay for detection of *Listeria* sp. target gene, using S1R and S1F oligonucleotides. DNA fragments amplified from kani-kama extraction. Wells with C+ and C- are the positive and negative controls respectively, the other wells with numbers correspond to the kani-kama samples that were positive and have (890bp). Well L corresponds to the 100bp molecular weight marker (Invitrogen®).
